# Cardiac repair in a murine model of myocardial infarction with human induced pluripotent stem cell-derived cardiomyocytes

**DOI:** 10.1186/s13287-020-01811-7

**Published:** 2020-07-17

**Authors:** Xin Jiang, Ziyi Yang, Ming Dong

**Affiliations:** 1 Department of Geriatrics, The Second Clinical Medical College of Jinan University, Shenzhen, 518020 Guangdong China; 2 Bioisland Laboratory, Biomedical Equipment Department, Building 3, No.188 KaiYuan Road, Huangpu District, Guangzhou, Guangdong China

**Keywords:** Induced pluripotent stem cells, Cardiomyocytes, Myocardial infarction

## Abstract

**Background:**

Cellular replacement strategies using human induced pluripotent stem cells (iPSCs) and their cardiac derivatives are emerging as novel treatments for post-myocardial infarction (MI) heart failure (HF); however, the mechanism of recovery of heart function is not very clear. The purpose of this study was to investigate the efficiency of using highly purified human induced pluripotent stem cell-derived cardiomyocytes (iPS-CMs) for myocardial repair in a mouse model of MI and to clarify the mechanism of recovery of heart function.

**Methods:**

Animals modelling MI were randomly assigned to receive direct intramyocardial injection of culture medium (MI group) or 4 × 10^5^ iPS-CMs (cell group) at the infarct border zone. Left ventricle (LV) performance was assessed with serial cardiac electrophysiology and was measured 1, 2 and 4 weeks post-MI. Invasive LV pressure measurement was measured at 4 weeks and was followed by sacrifice for histological examination.

**Results:**

Compared to the MI group, the left ventricle ejection fraction (LVEF), left ventricular internal diameter in end-diastole (LVIDd) and end-systole (LVIDs) and maximal positive and negative pressure derivative (±dP/dt) were significantly improved in the iPS-CM group at 4 weeks post-MI. Histological examination revealed a very limited number of iPS-CMs 4 weeks after transplantation. Nonetheless, there was a significant enhancement of angiogenesis and a reduction in apoptosis of native cardiomyocyte after iPS-CM transplantation.

**Conclusions:**

Our results demonstrate that transplantation of human iPS-CMs can improve heart function via paracrine action in a mouse model of myocardial infarction.

## Background

Myocardial infarction (MI) is a disease with high global mortality and morbidity rates. Permanent loss of cardiomyocytes (CMs) during MI contributes to progressive pathological left ventricular (LV) remodelling and heart failure (HF). Cell-based therapies using embryonic stem cells (ESCs) or induced pluripotent stem cells (iPSCs) and their cardiac derivatives have been proposed as an effective therapeutic approach to improve cardiac function in post-MI HF. ESCs and iPSCs are pluripotent stem cells that possess the ability to produce a sufficient amount of functional cardiomyocytes for “true” heart regeneration. Unlike ESCs, iPSCs are generated from an individual’s own somatic cells. Thus, iPSC isolation has fewer ethical concerns, and immunosuppression is not needed after transplantation. iPSCs might overcome some of the limitations of human ESCs for heart regeneration. Indeed, iPSCs can be differentiated into functional cardiomyocytes both in vitro and in vivo [1, 2]. A few papers have reported the functional benefit of using these iPS-derived cardiomyocytes (iPS-CMs) in transplantation for heart regeneration in rat or pig MI models. After transplantation, iPS-CMs were able to actively integrate with the host myocardium and improve LV function [3–6]. However, iPSC differentiation is in the initial stage, and the mechanism by which these cells improve heart function is not very clear. In this paper, we investigate the efficiency of using highly purified iPS-CMs for myocardial repair in a mouse model of MI and hope to clarify the mechanism of heart function improvement.

## Methods

### iPS-CM preparation

Human iPS-CMs were purchased from Cellular Dynamics International (CMC-100-010-001, CDI, USA); > 4.0 × 10^6^ viable cells were provided in cryovials. iCell cardiomyocytes labelled with green fluorescent protein (GFP), also from CDI, are highly purified human cardiomyocytes derived from iPSCs (> 90%) according to CDI’s proprietary differentiation and purification protocols. Cells were shipped as cryopreserved suspensions of dissociated cells with iCell Cardiomyocytes Plating Medium and iCell Cardiomyocytes Maintenance Media. iCell cardiomyocytes were maintained in liquid nitrogen until further use to ensure optimal performance. Before injection, cells were thawed and suspended in culture medium at a concentration of 4 × 10^5^/300 μl. iPS-CMs were characterised via the expression of the cardiac special marker troponin-T (1:200, Santa Cruz, CA). Cardiomyocytes were also assessed for senescence before transplantation with a β-Galactosidase Staining Kit (Cell Signaling Technology, #9860) according to the manufacturer’s instructions.

### Mouse model of MI and cell transplantation

**Fig. 1:**
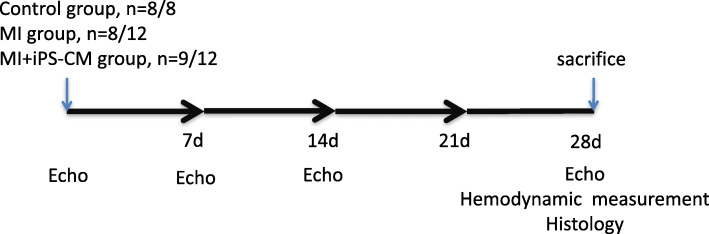
Experimental design and protocols. Four mice in the MI group and 3 mice in the iPS-CM group died during 4 weeks of observation after MI induction. At least 8 animals in each group survived for the duration of the experiment. There was no significant difference in mortality rate between these two groups

All animal experimental procedures were approved by the Committee on the Use of Live Animals in Teaching and Research at the University of Shenzhen. Adult male Imprinting Control Region (ICR) mice (12–16 weeks) underwent left anterior descending (LAD) coronary artery ligation to induce MI, as described previously [7]. Briefly, animals were anaesthetized with an intraperitoneal injection of ketamine 100 mg/kg and xylazine 20 mg/kg and then were connected to a mouse ventilator via tracheal intubation. Acute myocardial infarction was induced by LAD ligation in the middle of the left side by 8-0 sutures introduced via left thoracotomy. A successful MI model was confirmed by myocardial blanching at the apex. Ten minutes after ligation, animals were randomised to receive direct intramyocardial injections of 30 μl of (1) culture medium (MI group, *n* = 12) or (2) 4 × 10^5^ iPS-CMs (cell group, *n* = 12) at 3 different LV sites near the infarct border area. Another group of mice (*n* = 10) that served as controls underwent thoracotomy without coronary artery ligation (control group). Different groups in the study were subjected to invasive haemodynamic assessment 4 weeks post-MI and then were sacrificed for histological evaluation. The experimental design and protocols performed in the study are summarised in Fig. 1.

### Heart function evaluation by echocardiography

Echocardiography evaluations were performed using a Vevo 2100 high-resolution in vivo microimaging system equipped with a real-time microvisualisation scan head of 17.5 MHz (VisualSonics, Toronto, Ontario, Canada). An experienced operator who was blinded to the animal model and treatment performed all echocardiography. In brief, mice were anaesthetized via intraperitoneal injection of ketamine and xylazine as described above, and then the left lateral decubitus position on the anterior chest wall was examined. Standard M-mode parameters, including left ventricular internal diameter in end-diastole (LVIDd) and end-systole (LVIDs), posterior wall thickness (LVPW) and intraventricular septum thickness (IVS), were measured to calculate left ventricular ejection function (LVEF) according to the American Society of Echocardiography recommendations [8]. The mean value of three different cardiac cycles was used for each individual mouse at each time point. Serial echocardiography was performed to assess LV function on days 7, 14 and 28 after transplantation.

### Invasive cardiac haemodynamic assessment

Before sacrifice, mice were anaesthetized and mechanically ventilated as described above. A 1.4-Fr Millar catheter was inserted into the LV cavity via the right carotid artery and then was connected to a pressure transducer. Haemodynamic parameters, including maximal positive and negative pressure derivative (dP/dt), LV end-diastolic pressure (LVEDP) and LV end-systolic pressure (LVESP), were recorded with a PowerLab system (AD Instruments, Inc., Colorado Springs, CO).

### Histological evaluation

Hearts were harvested from animals 4 weeks after MI. The mRNA levels of PAI-1, MMP-3 and IL-6 were evaluated by RT-PCR to assess senescence in the control, MI and MI+ iPS-CM groups. Primer sequences for those mRNAs were as follows: PAI-1: 5′ primer- GACACCCTCAGCATGTTCATC and 3′ primer-AGGGTTGCACTAAAC ATGTCAG; MMP-3: 5′ primer-TTTAAAGGAAATCAGTTCTGGGCTATA and 3′ primer-CGATCTTCTTCACGGTTGCA; and IL-6: 5′ primer-ACACATGTTCTCTGGG AAATCGT and 3′ primer-AAGTGCATCATCGTTGTTCATACA. Other tissues were fixed with 10% buffered formalin and embedded in paraffin. Then, paraffin blocks were sectioned into 5-μm slides for haematoxylin and eosin (H&E) staining and immunofluorescence and immunohistochemical evaluation. The size of the infarct area was analysed using a Masson Trichrome Stain Kit (#HT15, Sigma). To assess the survival of engrafted cells and cardiac cell differentiation, cells were immunofluorescently labelled to detect GFP (green) and cardiac troponin-T (red) (1:200; Lab Vision, Fremont, CA) with double staining in tissues, and then they were visualised under a fluorescence microscopy. Staining with polyclonal rabbit anti-von Willebrand factor (vWF; AB7356, 1:200, Chemicon, Rosemont, IL) was used to measure capillary density in the infarct border area after cell transplantation. An in situ apoptotic cell death detection kit, POD (Roche Applied Science, Mannheim, Germany), which is based on the terminal deoxynucleotidyl nick end-labelling (TUNEL) assay, was used to evaluate apoptosis after cell transplantation. Quantitative analysis of positive vessels and apoptotic nuclei was performed in different sections at nine random fields from the infarct area and infarct border area in each animal. Each sample slice was photographed under a microscope (Olympus BX51, Olympus, Tokyo, Japan).

### Statistical analysis

Continuous variables are expressed as the mean ± SEM. A paired sample *t* test was used to compare 2 groups, and comparisons of variables between multiple groups were performed using one-way ANOVA. Calculations were performed with SPSS (ver. 16.0, Chicago), and *P ≤ 0*.05 was considered statistically significant.

## Results

### Animal survival

Four mice in the MI group and 3 mice in the iPS-CM group died during the 4 weeks of observation after cell transplantation. There was no significant difference in mortality rate between these two groups (Fig. 1, 4/12, 33% vs. 3/12, 25%, *P >* 0.1). We also found that among the total number of deaths, nearly 86% of mice died in the first week after MI (6/7). At the end of the experiment, there were at least 8 surviving animals in each group.

### Immunohistochemistry of single human iPS-CMs

**Fig. 2:**
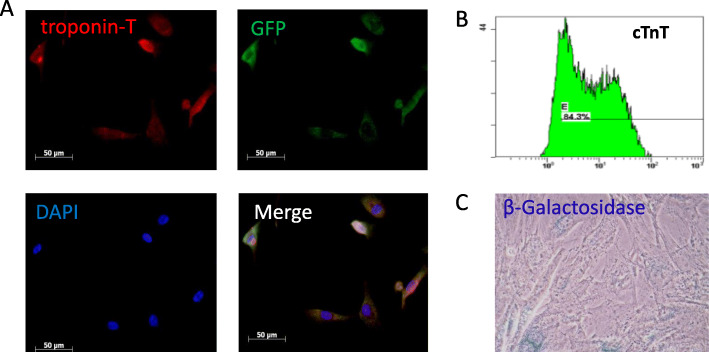
Characterisation of iPS-CMs via the expression of the cardiac special marker troponin T. **a** Cardiac cells were stained for troponin T with red, GFP with green, and DAPI with blue; a merged image is also shown. **b** Flow cytometry analysis of cell viability using anti-cardiac troponin T. The results showed that the percentage of iPS-CMs was approximately 85%. **c** β-Galactosidase staining did not show significant changes in cellular senescence

Flow cytometry was used to analyse cardiac troponin T in iPS-CMs. The results showed that the viability of cells in all batches was greater than 90% before transplantation, and the quality of differentiated cells was consistent among each batch. The identity of the CMs was verified by immunostaining for green fluorescent protein (GFP, green colour) and troponin T (red colour). The results from immunostaining and flow cytometry demonstrated that 85% of the total cells in the culture medium were iPS-CMs (Fig. 2b). β-Galactosidase staining did not show significant cellular senescence (blue colour, Fig. 2c).

### Cardiac function improvement after transplantation

**Fig. 3:**
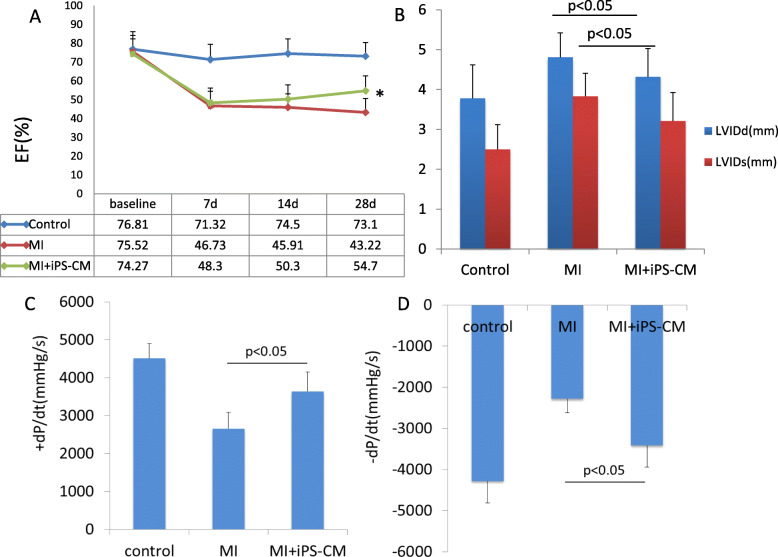
Cardiac function improvement after cell transplantation. **a**, **b** Left ventricle ejection fraction (LVEF), left ventricular internal diameter in end-diastole (LVIDd) and end-systole (LVIDs) values were measured by echo at baseline and 1 week, 2 weeks and 4 weeks post-MI among the three groups. Cardiac function was only improved significantly in the iPS-CM groups compared with the MI group at 4 weeks after cell transplantation. **c**, **d** Maximal positive and negative pressure derivatives (±dP/dt) were assessed by invasive haemodynamic measurement and showed significant improvement in the iPS-CM groups compared with the MI group at 4 weeks after cell transplantation

Heart function was assessed by a series of echocardiographic measurements collected on the 7th, 14th and 28th days following transplantation. On day 28, LVEF was significantly enhanced in the iPS-CMs group (54.7 ± 6.42%, *n* = 9) compared with the MI group (43.22 ± 7.71%, *n* = 8, *P* < 0.05; Fig. 3a); LVIDs and LVIDd values in the iPS-CMs group (4.32 ± 0.71 mm and 3.21 ± 0.72 mm) were significantly lower than those in the MI group (4.81 + 0.61 mm and 3.83 + 0.58 mm, *P* < 0.05; Fig. 3b). We did not observe any significant alterations in LVEF, LVIDs or LVIDd values between these two groups on the 7th and 14th days post MI. Invasive haemodynamic measurement at 4 weeks after cell transplantation revealed that iPS transplantation improved both +dP/dt (3638.9 ± 507.3) and −dP/dt (− 3415.5 ± 526.1) in MI (2657.64 ± 426.25 and − 2283.47 ± 338.73, *P* < 0.05, Fig. 3c, d).

### Cellular senescence evaluation

**Fig. 4:**
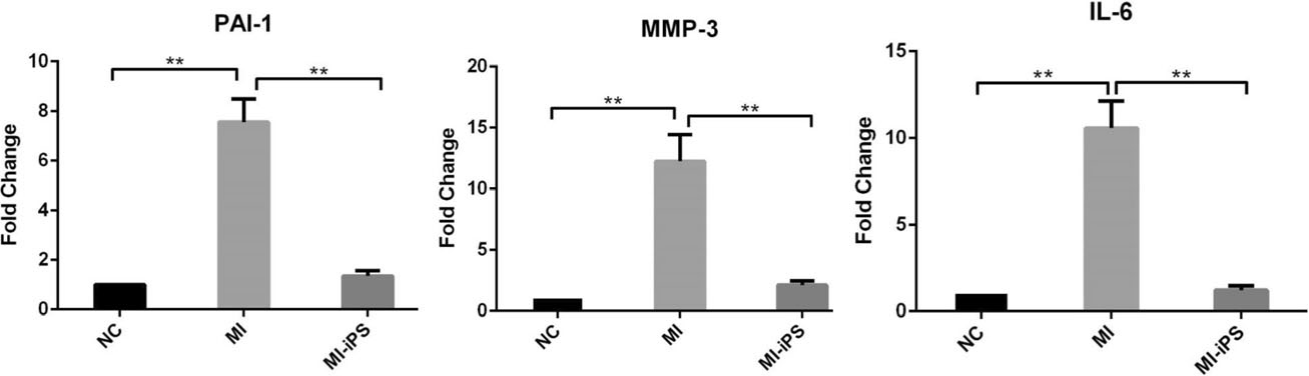
Cellular senescence improvement after cell transplantation. RT-PCR showed that the mRNA levels of PAI-1, MMP-3 and IL-6 were significantly increased in the MI group compared with the control group, and they significantly decreased after iPS-CM transplantation

Compared to their levels in the control animals, the animals subjected to the MI operation exhibited significantly elevated PAI-1, MMP-3 and IL-6 expression at the transcriptional level, which was attenuated by iPS transplantation. The mRNA levels of PAI-1, MMP-3 and IL-6 in the iPS-CM group were identical to those in the normal control group (Fig. 4).

### Immunohistochemical assessment of the infarct area

**Fig. 5:**
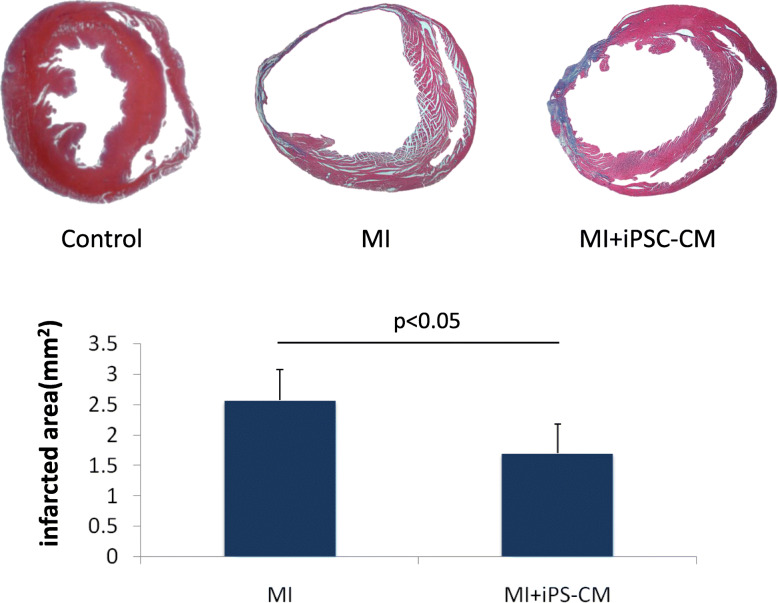
Infarct size reduction 4 weeks after cell transplantation. Masson trichrome staining showed that the infarct area (blue colour, *P* < 0.05) in the iPS-CM group was significantly smaller than it was in the MI group

Tissue sections were prepared and processed by Masson’s trichrome staining 4 weeks after transplantation (Fig. 5). The iPS-CM group had a smaller infarcted area than the MI group (2.57 ± 0.52 mm^2^ vs. 1.7 ± 0.48 mm^2^, *P* < 0.05). We did not observe any excessive proliferation of transplanted iPS at the injection areas or other positions throughout the myocardial tissue.

### iPS-CM engraftment and survival after transplantation

**Fig. 6:**
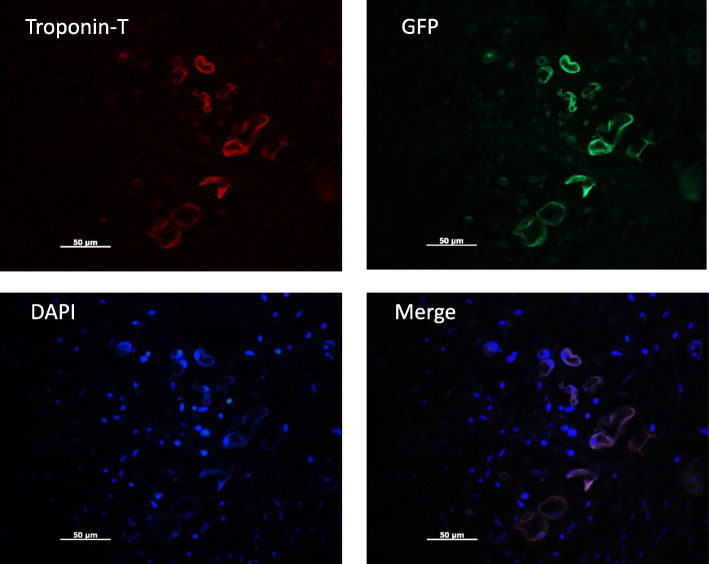
iPS-CM engraftment and survival after cell transplantation. Surviving transplanted cells were stained with troponin T (red), GFP (green), and DAPI (blue) at the infarct border area 4 weeks after transplantation; a merged image is also shown

Transplanted cells were detected by immunostaining for GFP and troponin T in tissue sections at the end of the 4th week after transplantation (Fig. 6). Less than 1% of cells in the myocardial tissues survived after 4 weeks of transplantation, as shown by quantification of GFP-positive cells, which were mainly observed in the infarct border area of animals in the cell group. The number of GFP-positive cells at this point was drastically decreased compared with day 7 (13%).

### Angiogenesis and decrease in apoptosis after transplantation

**Fig. 7:**
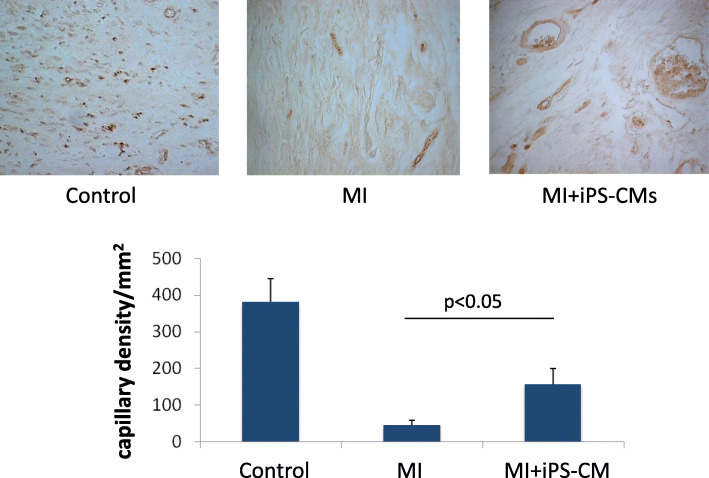
Angiogenesis after cell transplantation. Anti-von Willebrand factor (vWF) staining showed that the capillary density (brown colour) at the infarct border area was significantly higher in the iPS-CM group than it was in the MI group 4 weeks after transplantation

**Fig. 8:**
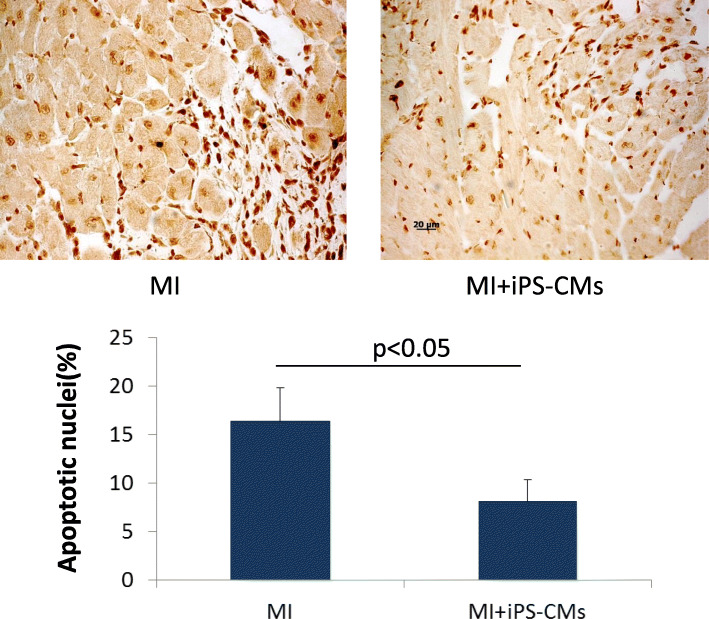
Anti-apoptosis after cell transplantation. Terminal deoxynucleotidyl nick end-labelling (TUNEL) staining revealed that there was a lower percentage of apoptotic cells at the infarct border area in the iPS-CM group than there was in the MI group 4 weeks after transplantation

The capillary density in the cell group (156.7 ± 42.3/mm^2^) was significantly higher than it was in the MI group (44.5 ± 13.4/mm^2^), as detected by vWF staining (*P* < 0.05, Fig. 7). Furthermore, TUNEL staining revealed a significantly lower percentage of apoptotic cells at the infarct border area in the cell transplantation group (21.93 ± 5.36%) than in the MI group (41.63 ± 8.29%, *P* < 0.05, Fig. 8).

## Discussion

There are two major types of stem cells that have the potential for heart regeneration: multipotent stem cells (adult stem cells) and pluripotent stem cells (ESCs, embryonic stem cells or iPSs). However, both types of cells present high risks of teratoma formation and heterotopic differentiation after transplantation into the host’s heart [9, 10]. To avoid this problem, differentiated cells, such as stem cell-derived cardiomyocytes, are preferred in stem cell therapy. Recent preclinical [11–13] and clinical studies [14] have demonstrated the great beneficial effect of human ESC-derived cardiomyocytes (ESC-CMs) and iPS-CMs in LV remodelling. Furthermore, iPS-CMs are autologous cells that are generated from somatic cells. Thus, there are fewer ethical concerns and a decreased need for immunosuppression after transplantation related to treatment with iPS-CMs compared with ESC-CMs. iPS-CMs should provide more predictable therapeutic effects and abilities for “off-the-shelf” use. However, the mechanism by which iPS-CMs improve heart function is not clear. In this paper, we found that transplantation of iPS-CMs produced therapeutic benefits that improved heart function and reduced infarct size in a mouse MI model. This effect was associated with angiogenesis and suppressed apoptosis of native cardiomyocytes in ischaemic cardiac tissue, which indicated that the mechanism of functional improvement was mediated by paracrine effects. These data have important clinical implications for the development of iPS-based therapies.

In this study, purified human iPS-CMs (> 85% pure in culture medium) were injected into the myocardium directly after MI. Four weeks after transplantation, the EF and dP/dt of the iPS-CM group improved significantly and there was a smaller infarct area than there was in the control group. Cell engraftment after transplantation was identified by GFP immunostaining. A small number of cells (< 1%) could be observed on the 4th week after transplantation into myocardium tissues. Therefore, it is unlikely that the transplanted cells directly contributed to the improvement of cardiac function. However, we found a significant increase in capillary density and a reduction in native CM apoptosis in the heart tissues following injection of cells. The transplanted cells enhanced angiogenesis and preserved the survival rate of the surrounding residential CMs via paracrine effects, which may ultimately lead to functional improvement. Furthermore, we did not observe any teratomas on any region of the heart.

Despite the great potential of iPSCs in regenerative medicine and cell therapy, there are several major hurdles that need to be addressed prior to their clinical application. First, the protocol using viral vectors to produce iPSCs may result in mutagenesis or malignant transformation. It is important to develop methods for reprogramming iPSCs without the need for any genetic modification and to obtain high purity (100%) and a high volume (10–100 billion) of functional cardiomyocytes prior to clinical application; such methods could involve single small molecules or virus-free methods [15, 16]. Second, safety evaluations, including arrhythmogenesis and tumorigenesis, are important concerns in cell-based therapy. Existing efforts mostly focus on cardiac differentiation, and limited attention has been paid to the important fact that transplanted CMs also need to exhibit a mature phenotype similar to that of the host myocardium. The immature electrical phenotypes of these cells might contribute to proarrhythmias. Proarrhythmic effects have been reported in ESC-CM graft studies [11, 17]. iPS-CMs also exhibit immature electrophysiological properties compared with mature adult CMs [18, 19]. However, the risk of arrhythmias following hESC-CM transplantation in injured hearts has not been determined. Future studies are needed to improve the maturation of these iPS-CMs before transplantation. Tumorigenesis is also an important concern in cell type choice. In this study, we did not observe any teratoma formation because CMs are a terminal stage of iPS and have limited ability to proliferate. Multilineage-differentiating stress enduring (Muse) cells are nontumorigenic endogenous pluripotent-like stem cells that can be obtained from various tissues, including the bone marrow, and they have shown long-lasting tissue repair and functional recovery after acute myocardial infarction in a rabbit model [20]. This may provide another selectable safe cell type for MI treatment.

Third, the mechanism by which cell transplantation improves cardiac function improvement is still not clear. The initial promise was that these transplanted stem cells could directly contribute to heart regeneration. However, recent studies proved that most of their beneficial effects are attributed to indirect actions, including paracrine actions, and modulation of the extracellular matrix and apoptosis, which is very similar to the results of this study. Unfortunately, which factors or proteins are the key contributors to the improvement of cardiac function has not yet been determined. Finally, one of the urgent problems of cell therapy is the low survival rate and engraftment after transplantation [21]. A recent study using human iPS-CMs for transplantation in a mini-pig MI model showed that only a small number of iPS-CMs were detected by fluorescence in situ hybridization at the 8th week after transplantation, indicating poor long-term survival and engraftment of the cells [22]. This progressive loss of transplanted cells could be a result of allogeneic immune rejection after transplantation. As an alternative to direct cell replacement, the release of exosomes from iPSC-CMs represents a new type of iPSC-based cardiomyogenesis therapy for heart regeneration [23]. These exosomes are known to possess anti-apoptotic effects, stimulate angiogenesis, reduce infarct size, and improve cardiac recovery. Positive outcomes of this study are associated with the secretion of paracrine factors in the absence of adequate cell survival, which may be mediated by the release of exosomes. Compared with those derived from iPSC-CMs alone, exosomes derived from MSCs cocultured with iPSC-CMs promoted longer survival and enhanced therapeutic effects in cells after transplantation [24]. The beneficial effects of exosomes secreted by iPSC-CMs in cardiac regeneration should be clarified in future studies.

This study provides support for the application of iPS-CM therapy for heart regeneration. We clarified the mechanism of heart function improvement after cell transplantation, which is a paracrine effect. Strategies to improve maturation, cell survival and therapeutic efficacy after transplantation require further investigation.

## Conclusions

Our results demonstrate that transplantation of human iPS-CMs can improve heart function via their paracrine effect in a mouse model of myocardial infarction.

### Study limitations and future perspectives

There are several limitations in this study. First, allogeneic murine model will induce immunoreaction after human cell transplantation, cell loss during the injection and immunorejection might have affected the experimental outcomes. The arrhythmogenesis after cell transplantation cannot be evaluated due to the high intrinsic beating rate and small size of the mouse heart. Future study using a large animal transplantation model receiving immunosuppression should allow better resolution for detailed functional and safety assessments. The optimal strategies of cardiac tissue bioengineering to improve cell survival and therapeutic effects also remain to be addressed.

## Data Availability

The data that support the findings of this study are available from the corresponding author upon reasonable request.

## Abbreviations

iPS: Induced pluripotent stem cells
MI: Myocardial infarction
HF: Heart failure
iPS-CMs: Induced pluripotent stem cell-derived cardiomyocytes
LV: Left ventricle
LVEF: Left ventricle ejection fraction
LVIDd: Left ventricular internal diameter in end-diastole
LVIDs: Left ventricular internal diameter in end-systole
ESC: Embryonic stem cells
GFP: Green fluorescent protein
ICR: Imprinting Control Region
LAD: Left anterior descending
LVPW: Left ventricular posterior wall thickness
IVS: Intraventricular septum thickness
LVEDP: LV end-diastolic pressure
LVESP: LV end-systolic pressure
